# Preparation of Nano-TiO_2_-Modified PVDF Membranes with Enhanced Antifouling Behaviors via Phase Inversion: Implications of Nanoparticle Dispersion Status in Casting Solutions

**DOI:** 10.3390/membranes12040386

**Published:** 2022-03-31

**Authors:** Jie Zhang, Ming Zheng, Yun Zhou, Linlin Yang, Yuanyuan Zhang, Zhichao Wu, Guocong Liu, Junjian Zheng

**Affiliations:** 1 School of Chemistry and Materials Engineering, Huizhou University, 46 Yanda Road, Huizhou 516007, China; jieemma2016@foxmail.com (J.Z.); gcl_109@163.com (G.L.); 2 College of Life and Environmental Science, Guilin University of Electronic Technology, 1 Jinji Road, Guilin 541004, China; y13079788398@163.com (L.Y.); zhangyuanyuan0226@hotmail.com (Y.Z.); 3 Key Laboratory of Organic Compound Pollution Control Engineering, School of Environmental and Chemical Engineering, Shanghai University, 333 Nanchen Road, Shanghai 200444, China; zmshu@shu.edu.cn; 4 State Environmental Protection Key Laboratory of Soil Health and Green Remediation, College of Resources and Environment, Huazhong Agricultural University, 1 Shizishan Street, Wuhan 430070, China; zhouyun06@126.com; 5 State Key Laboratory of Pollution Control and Resource Reuse, School of Environmental Science and Engineering, Tongji University, 1239 Siping Road, Shanghai 200092, China; wuzhichao@tongji.edu.cn

**Keywords:** TiO_2_ nanoparticles, dispersion, stability, composite membrane, antifouling property

## Abstract

Titanium dioxide (TiO_2_) nanoparticles have been applied in membrane antifouling performance modification for years. However, the influence of TiO_2_ nanoparticle dispersion status during the blending process on membrane properties and the inner mechanism has seldom been focused on. Herein, we investigated the influence of the various dispersing statuses of TiO_2_ nanoparticles on membrane properties and antifouling performance by exploring various blending processes without changing the original recipe. Polyethylene glycol (PEG) was employed as a pore-forming agent during the membrane preparation process, and also as a pre-dispersing agent for the TiO_2_ nanoparticles via the steric hindrance effect. Compared to the original preparation process of the PVDF/TiO_2_ composite membrane, the pre-dispersing of TiO_2_ via PEG ensured a modified membrane with uniform surface pores and structures on cross-sectional morphologies, larger porosity and water permeability, and more negative zeta potential. The contact angle was decreased by 6.0%, implying better hydrophilicity. The improved antifouling performance was corroborated by the increasing free energy of cohesion and adhesion, the interaction energy barrier (0.43 KT) between the membrane surfaces and approaching foulants assessed by classic XDLVO theory and the low flux decline in the filtration experiment. A kinetics mechanism analysis of the casting solutions, which found a low *TSI* value (<1.0), substantiated that the pre-dispersion of TiO_2_ with PEG contributed to the high stability and ultimately favorable antifouling behaviors. This study provides an optimized approach to the preparation of excellent nano-TiO_2_/polymeric composite membranes applied in the municipal sewage treatment field.

## 1. Introduction

In the last few decades, membrane separation technologies have been viewed as one of the most prominent strategies through which to address water quality and scarcity issues in various fields, such as water desalination, ultrapure water production and wastewater treatment [1]. Membrane fouling, arguably a major hindrance to membrane-based systems application, deteriorates membrane performance in terms of selectivity and productivity, shortens membrane life span and increases energy consumption [2]. Targeting this thorny problem, membrane modification, focusing on surface and/or bulk hydrophilicity improvement, has been acknowledged as a prominent approach for fouling mitigation, based on the consensus that hydrophilicity favors the amelioration of membrane fouling [3]. Modification strategies, such as surface coating [4], surface grafting [5] and the blending approach [6], have been practiced intensively. Among these, blending modification easily enables the incorporation of hydrophilic polymeric materials and/or nanoparticles into the membrane surface and bulk, which offers a window of opportunity for membranes to be modified via the synergy effect between polymers and hydrophilic compatible additives [7]. Organic materials and inorganic nanomaterials have frequently been blended to imbue membranes with desirable functional properties. Sandu et al. prepared microfiltration and ultrafiltration membranes by mixing acrylonitrile-vinyl acetate copolymers with poly (vinyl alcohol) [8]. Căprărescu et al. prepared a biopolymeric membrane by blending cellulose acetate with chitosan (CHI)-silver material to remove metallic ions [9]. Metal/metal oxide nanoparticles have received much attention in polymeric membrane modification in recent years [10]. Hanshim et al. modified a PVDF hollow-fiber membrane by using SiO_2_ particles as additives [11]. Căprărescu et al. synthesized a composite polyvinyl alcohol membrane with excellent proton conductivity by blending SiO_2_ nanoparticles [12]. Liang et al. modified the anti-irreversible-fouling performance of a polyvinylidene fluoride (PVDF) membrane by blending ZnO [13].

Among the inorganic metal oxide nanomaterials, TiO_2_ nanoparticles have attracted considerable attention for preparing composite membranes for years owing to their advantages of nontoxicity, low cost, superhydrophilicity and satisfactory chemical stability, as well as their antifouling and antibacterial properties [14]. In particular, the high water affinity of TiO_2_ nanoparticles, arising from the rich hydrogen bonding between water and surface hydroxyl groups, benefits membrane hydrophilicity modification to a large extent [15]. Nonetheless, the high surface energy of TiO_2_ nanoparticles inevitably gives rise to aggregation from their initial size (typically, around 20 nm) to several hundreds of nanometers, which is detrimental to their dispersion, exertion of hydrophilicity and self-cleaning properties in the membrane. To alleviate the negative influence of nanoparticle aggregation on membrane hydrophilicity modification, a growing body of literature has developed around complementary approaches for minimizing particle-to-particle interactions in the preparation of nanocomposite membranes. Zeng et al. prepared halloysite (HNTs)-loaded TiO_2_ and investigated the impact of TiO_2_-HNTs on the hydrophilicity property and antifouling performance of TiO_2_-HNTs/PVDF composite membranes [16]. Razmjou et al. used aminopropyl triethoxysilane (APTES) as a silane coupling agent to ease the aggregation of TiO_2_ nanoparticles, which improved the antifouling properties of a PES membrane [17]. Ma et al. modified the surface of TiO_2_ with -SO_3_H groups using sulfonated poly (phthalazinone ether sulfone ketone) (SPPESK) as a catalyst and, subsequently, prepared TiO_2_-SPEEK-PES nanocomposite membranes [18].

The modification of PVDF membranes adopting TiO_2_ nanoparticles has become a frequently discussed topic. The use of TiO_2_ was also proven to efficiently enhance hydrophilicity, water permeability and anti-fouling performance in our previous study [19]. Furthermore, extensive research has assessed how the incorporation of chemically functionalized/modified TiO_2_ nanoparticles affects the morphology, structure and performance of composite membranes. Nevertheless, little emphasis has been placed on the influence of the addition of TiO_2_ nanoparticles via the blending modification method without introducing additional chemical coating and changing the composition of the casting solution. The inner mechanism of TiO_2_ nanoparticles’ dispersion status during the blending process has not yet been clearly revealed. In this research, TiO_2_ nanoparticles were utilized as hydrophilic additives during a facile blending modification of a PVDF membrane, aiming at the application of this approach in a membrane bioreactor (MBR) in municipal sewage treatment. The dispersion of the TiO_2_ nanoparticles in casting solutions was implemented without changing the recipes simply by altering the manner of addition or sequence of TiO_2_ nanoparticles. The kinetics processes in the manifold casting solutions using an identical recipe were evaluated by multiple light scattering spectroscopy (MLiSSP). The influence on the membrane structure, properties and antifouling performance was examined systematically, aiming at preparing a PVDF/TiO_2_ composite membrane with excellent comprehensive properties conveniently. PEG was used beforehand in the casting solution preparation process to serve as the dispersant of TiO_2_ nanoparticles by virtue of its steric hindrance effects [20]. PEG can assist the pore-forming of composite membranes during the immersing phase inversion process; this is attributed to its capacity to form the hydration layer via hydrogen bonds that are relatively easy to break and reform [21]. The morphologies of the PVDF/TiO_2_ composite membranes fabricated by diverse manners of addition of nanoparticles were characterized by scanning electron microscope (SEM), while the cross-sectional elemental compositions of the membranes were determined by energy-dispersive X-ray (EDX). The membranes’ pore size distribution and mechanical properties were also estimated. The physicochemical properties of the membranes were explored by determining the contact angle, zeta potential, porosity and pure water permeability. The functional groups on the membrane surfaces were examined by attenuated total reflectance-Fourier transform infrared (ATR-FTIR). The membranes’ antifouling performance was simulated by extended Derjaguin–Landau–Verwey–Overbeek (XDLVO) theory and evaluated by batch filtration experiment. The stabilities of the casting solutions were also surveyed by multiple light scattering equipment (Turbiscan).

## 2. Materials and Methods

### 2.1. Reagents

Commercial-grade PVDF (Mw = 670~700 kDa) was obtained from Solvay Corporation (Brussels, Belgium). Dimethysulfoxide (DMSO) used as the solvent and PEG-400, used simultaneously as the dispersing agent of nanoparticles and the pore-forming additive during immersing inversion process, were provided by Sinopharm (Shanghai, China). TiO_2_ nanoparticles with an average particle size of 21 nm and bovine serum albumin (BSA, 67 kDa) were purchased from Sigma-Aldrich (St. Louis, MO, USA). The phosphate-buffered saline (PBS, pH = 7.4) was obtained by dissolving buffer salts (0.020% KCl, 0.790% NaCl, 0.024% KH_2_PO_4_ and 0.142% Na_2_HPO_4_) in deionized (DI) water. In total, 1 g/L BSA with PBS solution at pH 7.0, adjusted by 0.1 M NaOH or 0.1 M HCl, was used as a model foulant in this study.

### 2.2. Membrane Preparation

**Figure 1:**
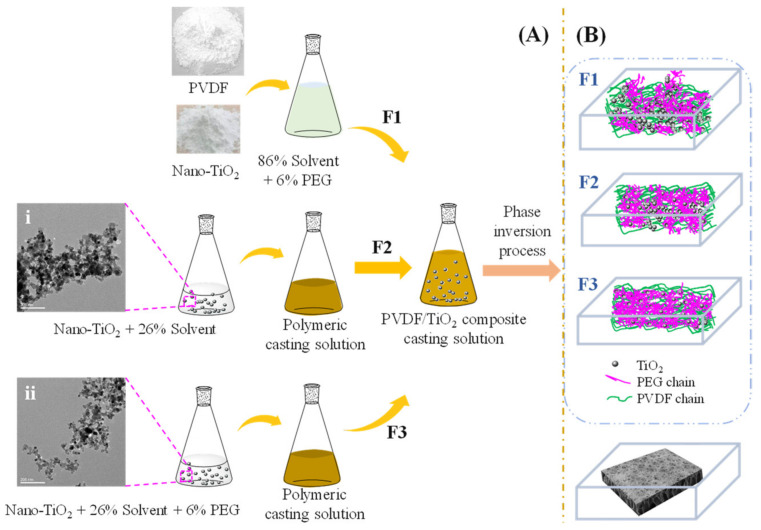
Schematic illustration mechanism for the preparation process of PVDF/TiO_2_ composite membranes F1–F3: (**A**) casting solution preparation process and (**B**) phase inversion process.

Preparation processes of PVDF/TiO_2_ composite membranes F1–F3 are shown in Figure 1. Dosages of PVDF material, DMSO, PEG and TiO_2_ nanoparticles were set as 8 wt.%, 86 vol.%, 6.0 vol.% and 0.15 wt.%, respectively. For preparing pristine PVDF/TiO_2_ composite membrane F1 via traditional process, TiO_2_ nanoparticles and PVDF material were added into the mixture of solvents and PEG additive. The composite solution was stirred by employing a mechanical stirrer at 80 °C (under oil-bath heating) for 7 d in a fume cupboard to obtain the final casting solution for membrane F1. To compare the effect of the absence and presence of PEG in the pre-dispersion step on the membrane morphologies and properties, the PVDF/TiO_2_ composite membranes were prepared via two strategies. First, 86 vol.% solvent was separated into two parts, i.e., 26 vol.% and 60 vol.%. Subsequently, TiO_2_ nanoparticles were dispersed in 26 vol.% solvent without adding PEG to form the suspension of membrane F2, while for membrane F3, 26 vol.% solvent and 6.0 vol.% PEG were jointly employed to disperse TiO_2_ nanoparticles. Next, the two suspensions were subjected to ultrasonication at 20 °C for 20 min and then added into the pre-prepared homogenous solutions containing resting components (60 vol.% DMSO and 8 wt.% PVDF and 6 wt.% PEG for membrane F2; 60 vol.% DMSO and 8 wt.% PVDF for membrane F3). This was followed by the dissolution of the mixed solutions at 80 °C for 4 d. The final casting solutions for membranes F2 and F3 were attained after the stirring of the aforementioned mixed solutions at 80 °C for another 3 d. The dispersion of TiO_2_ nanoparticles determined by transmission electron microscopy (TEM) and a schematic illustration mechanism for the preparation process of PVDF/TiO_2_ composite membranes F1–F3 are shown in Figure 1. All the membranes in this study were prepared by phase inversion via immersion precipitation method. The casting solutions were cast on porous polyester non-woven fabrics/flat glass plates with a casting gap of 250 μm. After evaporating to ambient air for 30 s, the casting films, together with fabrics/flat glass plates, were immersed in a coagulation bath (deionized water) at room temperature, during which PVDF/TiO_2_ composite membranes F1–F3 were formed.

### 2.3. Membrane Characterization

Membrane morphologies for both surfaces and cross-sections were determined by field emission scanning electron microscope (SEM, S-4800, Hitachi, Tokyo, Japan). The data of pore size and porosity on the surface were collected by Image-Pro Plus 6.0 software (Media Cybernetics, Rockville, MD, USA) and statistically analyzed using Microsoft Excel, as in our previous study [22]. Thickness of membranes with non-woven fabrics was measured by employing a micrometer at five different locations. Energy-dispersive X-ray (EDX) spectroscopy was employed to assess the elemental compositions and the dispersion of TiO_2_ on the cross-section of membrane. Overall membrane porosity, which can be calculated by Equation (1), was determined by gravimetric method at least three times.

(1) $$\varepsilon =\frac{m_{1}-m_{2}}{\rho_{w}\cdot A\cdot l}$$

where *m*_1_ and *m*_2_ are the weights of the wet and dry membranes (g), respectively. The value *ρ_w_* is the water density (1 g/cm^3^) and *A* is the effective area of the membrane (cm^2^). The value *l* is the membrane thickness (cm), which was determined by micrometer caliper at different areas of membrane surfaces five times.

Water permeability and mechanical properties were tested three times using the method reported in our previous study [22]. Membrane surface hydrophilicity characterized by contact angle was observed by optical measurement system (OCA 15 Plus, Data physics GmbH, Filderstadt, Germany). Zeta potential of membrane surface was determined by streaming potential analyzer (EKA 1.00, Anton-Paar, Graz, Swiss), during which 10 mM KCl solution with a pH value of 7.0 was used as flowing liquid. The functional groups on membrane surfaces were identified by attenuated total reflectance-Fourier transform infrared (ATR-FTIR, Thermo Electron Corporation, Waltham, MA, USA) with a resolution of 4 cm^−1^. The surface roughness of each membrane sample, determined as average roughness (*R*a), root-mean-square roughness (*R*q) and maximum roughness (*R*max) was determined by atomic force microscope (AFM, Dimension 5000, Bruker AXS, Santa Barbara, CA, USA) three times.

### 2.4. Antifouling Performance Evaluation

#### 2.4.1. XDLVO Theory Analysis

Contact angles determined by applying three probe liquids (water, formamide and diiodomethane) can be used to determine the membrane surface tension parameters by adapting extended Young’s Equations (2)–(4) [23].

(2) $$\gamma^{\mathrm{AB}}=2\sqrt{\gamma^{+}\gamma^{-}}$$

(3) $$\gamma^{\mathrm{TOT}}=\gamma^{\mathrm{LW}}+\gamma^{\mathrm{AB}}$$

(4) $$\left(1+\cos\theta \right)\;\gamma_{l}^{\mathrm{TOT}}=2\left(\sqrt{\gamma_{s}^{\mathrm{LW}}\gamma_{l}^{\mathrm{LW}}}+\sqrt{\gamma_{s}^{+}\gamma_{l}^{-}}+\sqrt{\gamma_{l}^{+}\gamma_{s}^{-}}\right)$$

where *γ*^+^ is the electron acceptor parameter, *γ*^−^ is the electron donor parameter and *θ* is the contact angle. The value *γ*^AB^ is the acid-base (AB) component of surface tension, *γ*^LW^ is the Liftshiz–van der Waals (LW) component of surface tension and *γ*^TOT^ is the total surface tension. The subscript (s) refers to either membrane surface or foulants (BSA in this study) and (l) indicates the probe liquid used in the measurements.

The free energy of adhesion between membranes and BSA per unit area can be calculated by Equation (5). The values Δ*G_h_*_0_^LW^, Δ*G_h_*_0_^AB^ and Δ*G_h_*_0_^EL^ denote LW, AB and electrostatic (EL) interaction free energy components at the minimum separation distance *h*_0_ (*h*_0_ ≈ 0.158 nm), which can be determined by Equations (6)–(8), respectively [24]. The free energy of cohesion for membranes and the corresponding components can also be obtained by Equations (5)–(8), when *γ*_c_ is replaced by *γ*_m_.

(5) $$\Delta G_{h_{0}}^{\mathrm{TOT}}=\Delta G_{h_{0}}^{\mathrm{LW}}+\Delta G_{h_{0}}^{\mathrm{AB}}+\Delta G_{h_{0}}^{\mathrm{EL}}$$

(6) $$\Delta G_{h_{0}}^{\mathrm{LW}}=2\left(\sqrt{\gamma_{l}^{\mathrm{LW}}}-\sqrt{\gamma_{m}^{\mathrm{LW}}}\right)\left(\sqrt{\gamma_{c}^{\mathrm{LW}}}-\sqrt{\gamma_{l}^{\mathrm{LW}}}\right)$$

(7) $$\Delta G_{h_{0}}^{\mathrm{AB}}=2\sqrt{\gamma_{l}^{+}}\left(\sqrt{\gamma_{m}^{-}}+\sqrt{\gamma_{c}^{-}}-\sqrt{\gamma_{l}^{-}}\right)+2\sqrt{\gamma_{l}^{-}}\left(\sqrt{\gamma_{m}^{+}}+\sqrt{\gamma_{c}^{+}}-\sqrt{\gamma_{l}^{+}}\right)-2\left(\sqrt{\gamma_{m}^{+}\gamma_{c}^{-}}+\sqrt{\gamma_{c}^{+}\gamma_{m}^{-}}\right)$$

(8) $$\Delta G_{h_{0}}^{\mathrm{EL}}=\frac{\kappa \varepsilon_{0}\varepsilon_{r}}{2}\left(\zeta_{c}^{2}+\zeta_{m}^{2}\right)\times \left(1-\coth(\right.\kappa h_{0})+\frac{2\zeta_{m}\zeta_{c}}{\left(\zeta_{c}^{2}+\zeta_{m}^{2}\right)}\mathrm{csch}\left.\left(\kappa h_{0}\right)\right)$$

where *ε*_r_ is the dielectric constant of water, *ε*_0_ is the dielectric permittivity of vacuum, *ε*_0_*ε*_r_ is the dielectric permittivity of the fluid, *κ* is the inverse Debye screening length, *ζ*_m_ is the surface potential of membrane, and *ζ*_c_ is zeta potential of BSA solution, respectively. The subscripts m, l and c denote membrane, bulk liquid (water in this study) and BSA, respectively. The inverse Debye screening length, *κ*, is determined by Equation (9).

(9) $$\kappa =\sqrt{\frac{e^{2}\sum n_{i}z_{i}^{2}}{\varepsilon_{r}\varepsilon_{0}kT}}$$

where *e* is the electron charge, *n_i_* is the number concentration of ion *i* in the bulk solution, *z_i_* is the valence of ion *i*, *k* is Boltzmann’s constant and *T* is the absolute temperature.

The LW, AB and EL interaction energy components between membrane and BSA (*U*_mlc_^LW^, *U*_mlc_^AB^ and *U*_mlc_^EL^) can be calculated through Equations (10)–(12), respectively. The summation of above interaction energy components expresses the total energy balance for aqueous systems (shown as Equation (13)).

(10) $$U_{\mathrm{mlc}}^{\mathrm{LW}}=2\pi \Delta G_{h_{0}}^{LW}\frac{h_{0}^{2}a}{h}$$

(11) $$U_{\mathrm{mlc}}^{\mathrm{AB}}=2\pi a\lambda \Delta G_{h_{0}}^{AB}\exp\left[\frac{h_{0}-h}{h}\right]$$

(12) $$U_{\mathrm{mlc}}^{\mathrm{EL}}=\pi \varepsilon_{0}\varepsilon_{r}a\left[2\zeta_{c}\zeta_{m}\ln\left(\frac{1+e^{-\kappa h}}{1-e^{-\kappa h}}\right)+\left(\zeta_{c}^{2}+\zeta_{m}^{2}\right)\ln(1-e^{-2\kappa h})\right]$$

(13) $$U_{\mathrm{mlc}}^{\mathrm{XDLVO}}=U_{\mathrm{mlc}}^{\mathrm{LW}}{}^{}+U_{\mathrm{mlc}}^{\mathrm{EL}}+U_{\mathrm{mlc}}^{\mathrm{AB}}$$

where *a* is the radius of BSA, *h* is the separation distance between membrane and BSA and *λ* is the decay length of AB interactions (~0.6 nm).

#### 2.4.2. Filtration Performance

To evaluate the membrane antifouling properties, a filtration experiment was carried out using a filtration cell (MSC300, Mosu Corporation, China) at room temperature. Membrane sample was pre-compressed by filtrating DI water for 30 min at 0.05 MPa and then filtrated by 250 mL BSA solution under a magnetic stirring rate of 500 rpm. The flux was recorded and calculated by Equation (14) every 25 s.

(14) $$J=\frac{m}{A\cdot \Delta t}$$

where *m* is the volume of permeated water (L), *A* is the effective membrane filtration area (m^2^) and Δ*t* is the permeation time (h).

### 2.5. Casting Solution Stability

Multiple light scattering spectroscopy (Turbiscan Tower, Formulaction, Toulouse, France) with near-infrared light source (*λ* = 880 nm) was operated at 80 °C for 12 h to delve into the kinetics mechanism in the nano-composited casting solutions, during which the real-time dynamic processes were monitored. The casting solutions were filled into glass tubes and then inserted into the chambers, followed by monitoring transmission (T) and backscattering (BS) signals by two detector devices, i.e., the transmission detector (0° from the incident light) and the backscattering detector (135° from the incident radiation) along the cell height [25]. Bottom of sample tubes was defined as 0 mm, and the height increased along with the tubes. Backscattering signal (Δ*BS*) referring to 0 h was analyzed in this study.

The stability of different casting solution samples can be indicated by a statistical factor, Turbiscan Stability Index (*TSI*), which can be captured as the sum of all processes occurring in the investigated systems [26]. The larger *TSI* value, the less stable the given system [27]. The *TSI* values can be obtained using Equation (15).

(15) $$TSI=\sqrt{\frac{\sum_{i=1}^{n}{\left(x_{i}-x_{BS}\right)}^{2}}{n-1}}$$

where *x_i_* means the average backscattering for each minute of measurement, *x_BS_* refers to the average *x_i_* and *n* is the number of scans.

## 3. Results and Discussion

### 3.1. Membrane Morphologies

**Figure 2:**
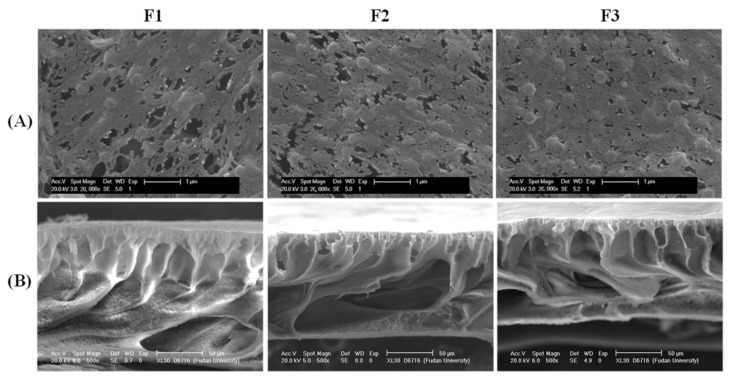
Surface morphologies (**A**) and cross-sectional morphologies (**B**) of PVDF/TiO_2_ composite membranes F1–F3.

**Figure 3:**
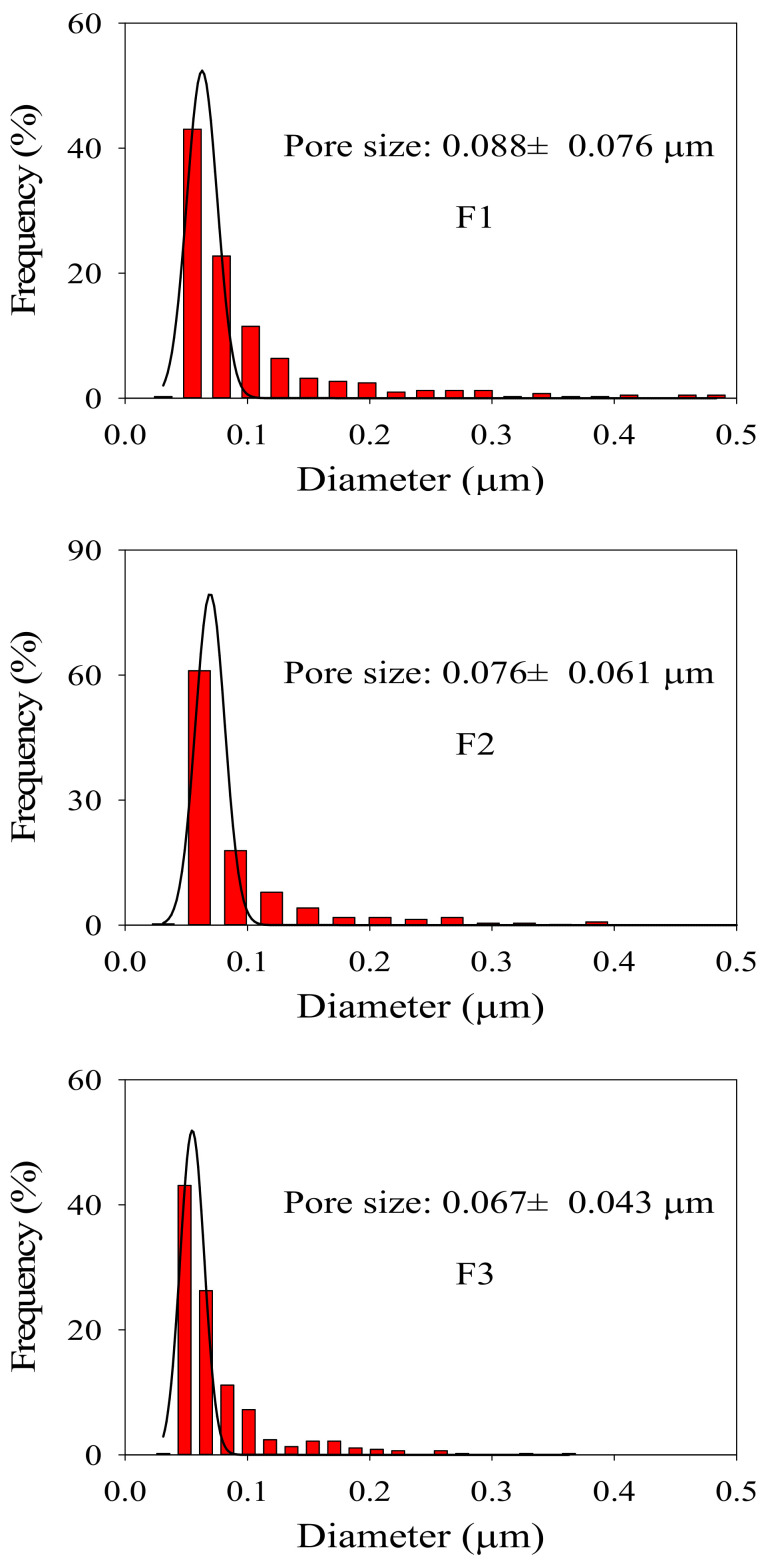
Pore size distribution histograms on the surfaces of PVDF/TiO_2_ composite membranes F1–F3.

**Figure 4:**
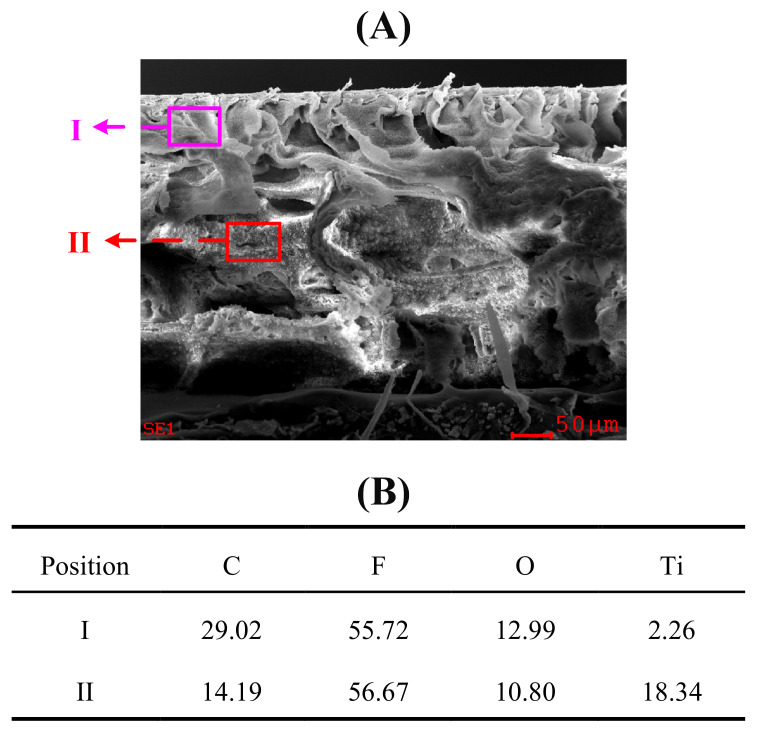
(**A**) Cross-sectional morphology of PVDF/TiO_2_ composite membrane F1 and (**B**) elemental composition (wt.%) of position I and II determined by EDX.

Figure 2A exhibits the surface morphologies of PVDF/TiO_2_ composite membranes F1–F3. The pore size on the surface of membrane F3 was smaller and more evenly dispersed than those of membrane F2 and membrane F1. In accordance with this observation, the average pore sizes for membranes F1–F3 were determined to be 0.088 ± 0.076 μm, 0.076 ± 0.061 μm and 0.067 ± 0.043 μm, respectively (see Figure 3), indicating decreasing pore sizes and increasing uniformity on the membrane surfaces. Moreover, no obvious aggregation of TiO_2_ nanoparticles was observed on the surface of membrane F3. This might be attributed to the fact that the hydrophilic PEG may have provided rich coordination sites for the TiO_2_ nanoparticles via hydrogen bonds together with steric hindrance effects, thereby promoting the dispersion of TiO_2_ nanoparticles in the composite casting solution and their immobilization in the formed film after the release of PEG during the immersing phase inversion process (see Figure 1B). As delineated in Figure 2B for the cross-sectional morphologies, membranes F1–F3 uniformly had a thin and dense skin layer, a porous sub-layer and finger-like macrovoids, but with diverse developed extents at the bottom. According to the kinetics of film formation, the exchange velocity between the solvent and non-solvent phase (water in this study) after immersion in a coagulation bath can significantly affect the development of macrovoids and membrane thickness [28]. As indicated in Figure 1B, the settlement under gravity of the aggregated TiO_2_ nanoparticles in the casting solution of membrane F1 might have accelerated the precipitation rate and decelerated the exchange velocity between the solvent and the nonsolvent, which hindered the development of microvoids and thus led to the development of a suppressed finger-like structure in comparison to membranes F2 and F3. Meanwhile, owing to the changed exchange velocity, the nano-TiO_2_-modified membranes were found to differ from the membrane without TiO_2_ addition in terms of thickness [20]. The elemental composition on different cross-sectional layers of membrane F1 is shown in Figure 4, which suggests that the majority of the TiO_2_ nanoparticles were deposited on the bottom macrovoid layer instead of either the skin layer or the sub-layer, as hypothesized. The pre-dispersion process under the steric hindrance effects of the PEG for membrane F3 contributed to the homogeneous dispersion of TiO_2_ nanoparticles both on the surface and the sub-layers and facilitated the development of a finger-like structure. Compared to the fully developed finger-like structure of membrane F2, the finger-like macrovoids of membrane F3 might have been restricted by the steric hindrance effects of the PEG in some way. Furthermore, the considerable PEG chains adhered to the TiO_2_ nanoparticles were assumed to increase the viscosity of the casting solution, impeding the free development of a finger-like structure for membrane F3 [29]. Hence, the proper dose ratio of TiO_2_/PEG needs to be further explored.

### 3.2. Membrane Properties

**Figure 5:**
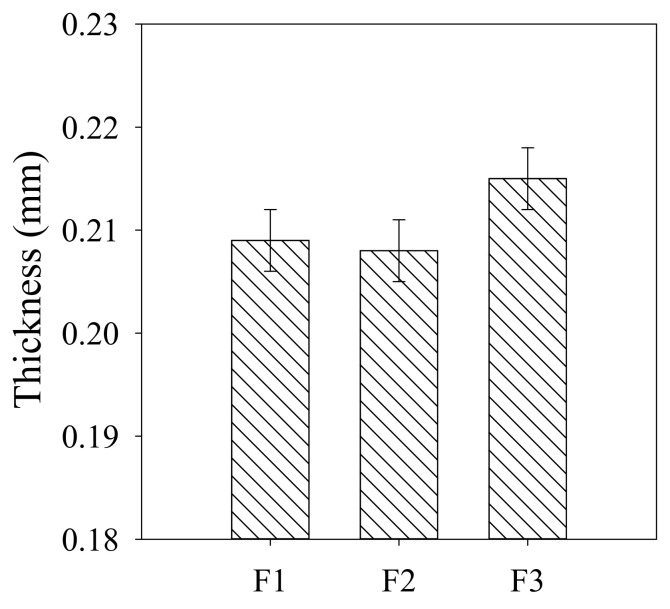
Thickness of asymmetric PVDF/TiO_2_ composite membranes F1–F3.

The thicknesses of the asymmetric PVDF/TiO_2_ composite membranes with non-woven fabrics measured by applying a micrometer are shown in Figure 5, based on which the determined porosity and pure water flux are exhibited in Table 1. With the pre-dispersion of the TiO_2_ nanoparticles, the porosity of membranes F2 and F3 was improved, which was likely attributable to the less closed and tortuous macrovoids along with the larger finger-like connected pores. However, the porosity of membrane F3 was lower than that of membrane F2, owing to the fully developed finger-like structure observed on the cross-sectional morphology of membrane F2. The water permeability of membranes F1–F3 was in accordance with the porosity results. As is known, porosity and surface pore size play significant roles in raising the permeability of membranes [16]. The permeability of membrane F3 was only slightly decreased compared to that of membrane F2, despite its smaller surface pore size and intricate cross-sectional structure. This might have been due to the evidently enhanced hydrophilicity of membrane F3 (see Table 1). Compared to membrane F1, the contact angles of membranes F2 and F3 decreased by 1.0% and 6.0%, respectively, suggesting the improvement in their hydrophilicity after the pre-dispersion of TiO_2_ nanoparticles during the membrane preparation process. In a similar vein, previous studies suggested that the contact angles of composite membranes were decreased by 2.8−18% following the addition of 0.15−6% of TiO_2_ nanoparticles [17,20]. The obviously improved hydrophilicity of membrane F3 was possibly also derived from the residual hydrophilic PEG chains, which were not easily released because of their entanglement with the TiO_2_ nanoparticles during the exchange process between the solvent and the nonsolvent. As shown in Table 1, the zeta potential of membranes F1–F3 showed the same tendency to that of the contact angle. For the membrane F3, with small pores, the TiO_2_ nanoparticles were supposed to form a layer uniformly on the membrane surface. However, for the membranes F1 and F2, with large pores, the TiO_2_ nanoparticles presumably entered into the membrane structure and became entrapped within the inner pores.

**Figure 6:**
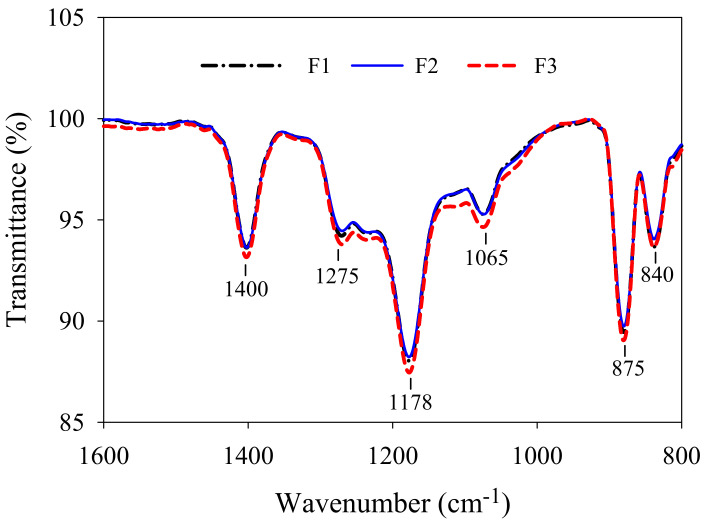
ATR-FTIR spectra of PVDF/TiO_2_ composite membranes F1–F3.

The mechanical strength of membranes F1–F3, characterized by tensile strength and elongation at break, are shown in Table 2. It can be observed that the tensile strength of membranes F1–F3 was reinforced gradually. The agglomeration of the TiO_2_ nanoparticles in the porous sub-layers might have resulted in poor compatibility within the polymer bulk, thus diminishing the modulus of membrane F1 [30]. The TiO_2_ nanoparticles acting as cross-linking points intensified the interaction of the polymeric chains in membranes F2 and F3, which meant that more energy was needed to conquer the interaction or break down the bond between them [31]. In the case of the same dosage of TiO_2_ nanoparticles, less agglomeration implied more cross-linking points, which was conductive to forming strong interactions throughout membrane bulk, and which, consequently, produced higher mechanical strength. The lower elongation at break for membrane F2 could be attributed to the relatively fully developed finger-like structure and larger connected macrovoids. As exhibited in Table 3, the roughness of membrane F1 was slightly higher than those of membranes F2 and F3, probably due to the agglomeration of TiO_2_ nanoparticles on its surface. The roughness of membranes F2 and F3 showed a marginal difference. Overall, the membranes F2 and F3 exhibited higher hydrophilicity, more negative zeta potential and lower roughness in comparison to membrane F1, implying their superior antifouling performance. The ATR-FTIR spectra results are shown in Figure 6. The peak at 1400 cm^−1^ was associated with the deformation vibration of -CH_2_, while those at 1275 cm^−1^ and 1178 cm^−1^ were associated with the symmetrical and asymmetrical stretching of -CF_2_, the peak at 875 cm^−1^ was associated with one of the characteristic peaks of the PVDF and the peak at 840 cm^−1^ was associated with the stretching vibration of -CH [32]. The peak at 1065 cm^−1^ was considered as the stretching vibration of -OH [22]. Membrane F3 showed higher intensity, indicating better hydrophilicity.

### 3.3. Membrane Antifouling Performance Evaluation

**Figure 7:**
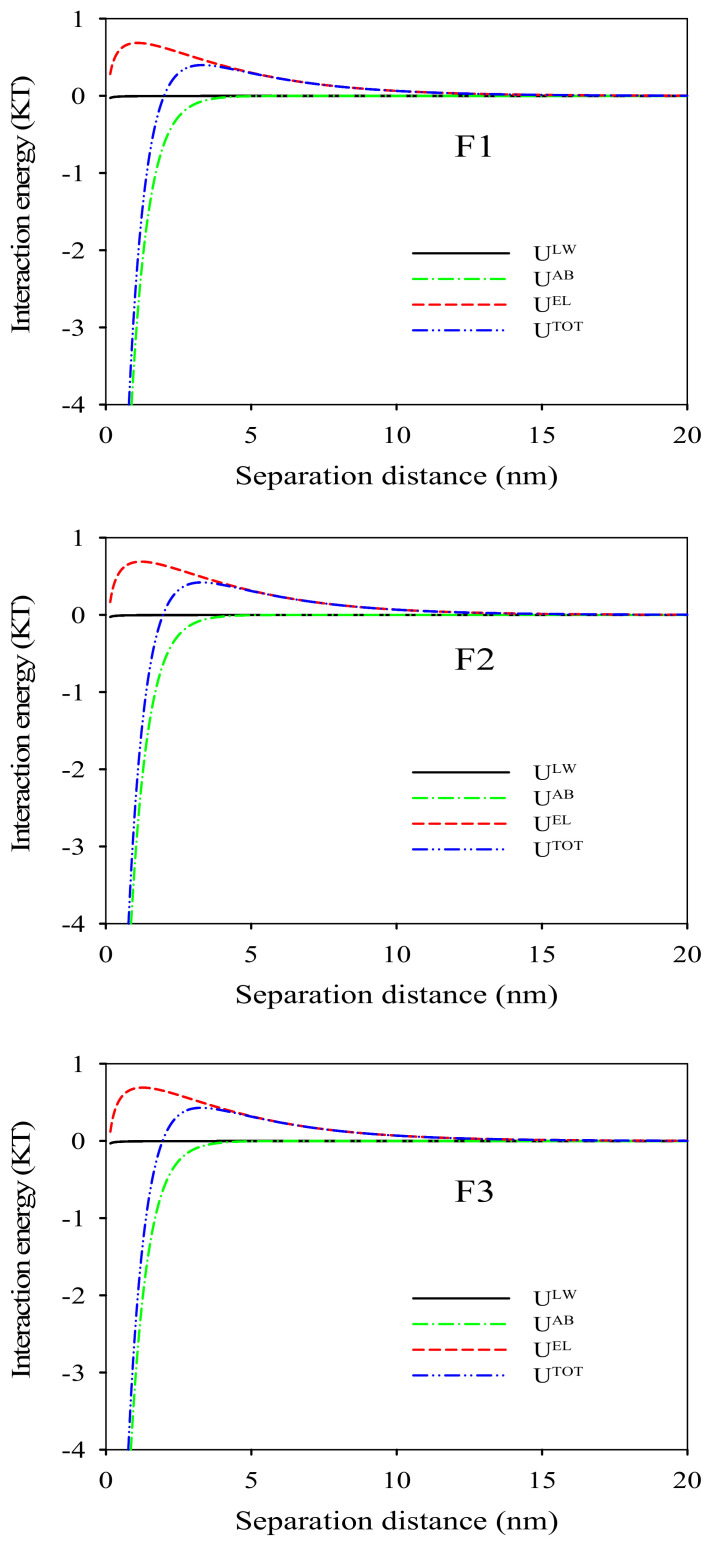
Variations of interaction energy components between BSA and the surface of PVDF/TiO_2_ composite membranes F1–F3 versus separation distance.

**Figure 8:**
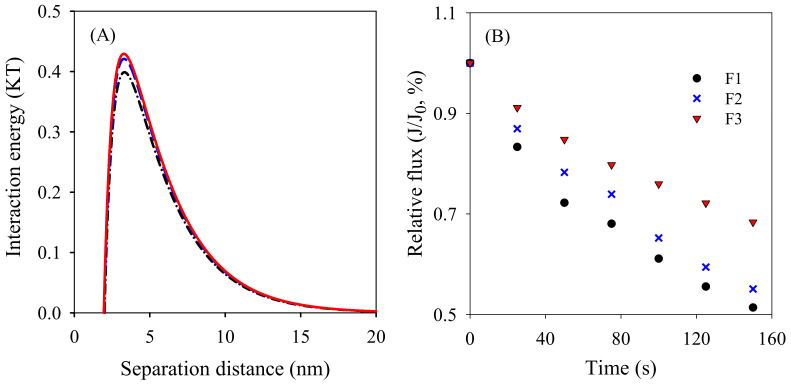
(**A**) Interaction energy between membrane surfaces and approaching foulants and (**B**) normalized flux of membranes when filtrating BSA solution.

The properties of BSA, zeta potential and contact angles of membranes F1–F3 determined by employing three probe liquids are displayed in Table 4. The surface tension parameters for each membrane and BSA are displayed in Table 5. The increasing electron donor parameter (*γ*^−^) was apparently due to the abundant hydroxyl groups presented on the TiO_2_ nanoparticles. This phenomenon proved the fact that more TiO_2_ nanoparticles were distributed on the surfaces of membranes F2 and F3 prepared by the pre-dispersion method. The negative free energy of cohesion of the membranes implied an attraction tendency. The more negative the value, the stronger the attraction tendency [24]. Following this logic, the decreasing negative free energy of cohesion (see Table 5 and Table 6) demonstrated the decreasing attraction tendency of the membrane surfaces. Similarly, the decreasing negative free energy of adhesion for membranes F1–F3 (see Table 5 and Table 6) manifested the decreasing attraction interaction between the membrane surfaces and the foulants, suggesting the increasing antifouling property. The interaction energy versus the approaching distances between the membrane surfaces and the foulants are shown in Figure 7 and Figure 8A. The positive interaction energy means the repulsive interaction between the membrane surfaces and the approaching foulants. The interaction energy peak for membrane F3 was 0.43 KT, which was higher than that of membranes F1 (0.40 KT) and F2 (0.42 KT) (shown in Figure 8A). The higher interaction energy peak implied the increasing difficulty with which the foulants were attached or settled on the membrane surfaces. The progressively increased interaction energy revealed the consistent pattern of the free energy of cohesion and adhesion, which clearly verified the progressively enhanced antifouling performance.

A batch filtration experiment was conducted to compare the antifouling performance of membranes F1–F3. As observed in Figure 8B, the flux of membrane F1 declined the fastest, followed by membranes F2 and F3. This result further confirms the satisfactory antifouling modification by preparing the PVDF/TiO_2_ composite membrane simply through the pre-dispersion of the TiO_2_ nanoparticles. The slightly decreased roughness on the surfaces of membranes F2 and F3 might also have contributed to the enhanced antifouling performance [33].

### 3.4. Stability of Casting Solutions for Membranes F1–F3

**Figure 9:**
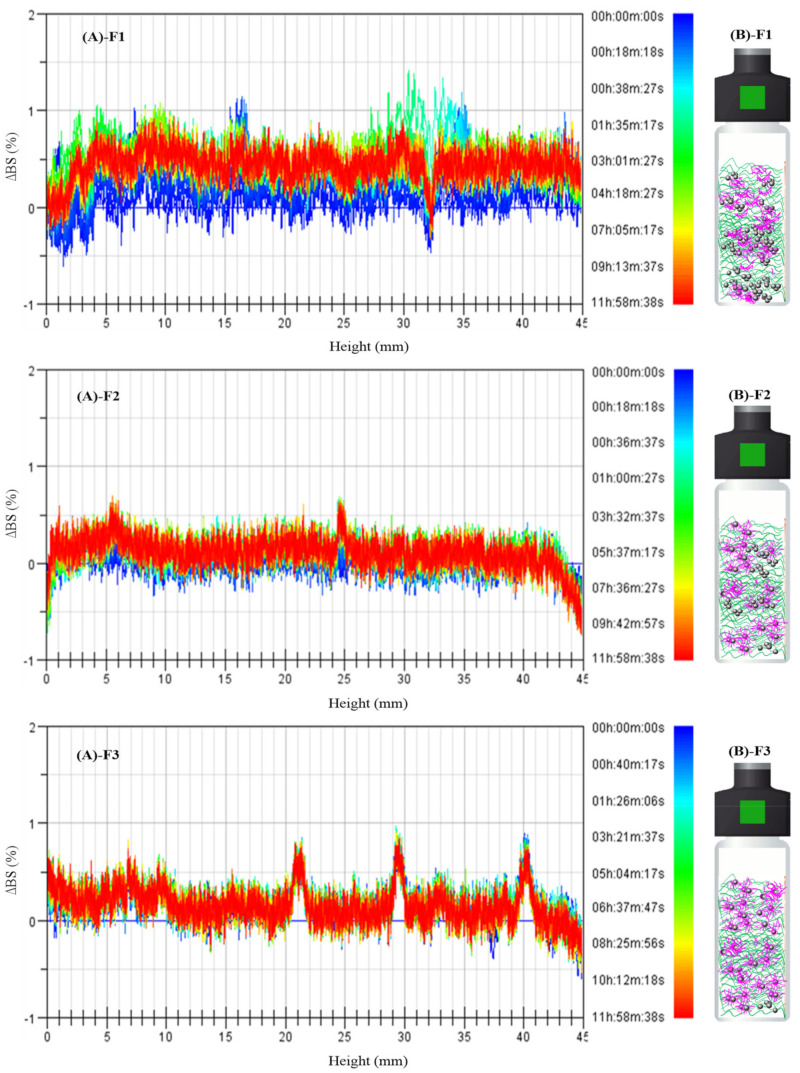
(**A**) Backscattering intensity profiles along the sample height and (**B**) schematic diagram for various casting solution samples F1–F3.

**Figure 10:**
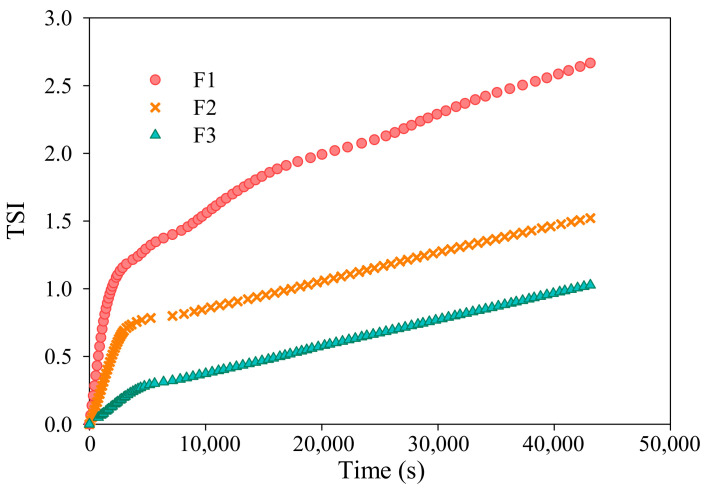
*TSI* of casting solutions of membranes F1–F3 throughout the measurement period of 12 h.

By virtue of Turbiscan equipment, the kinetics of nano-composited casting solutions can be clarified by measuring backscattering light and transmitted light signals. In this study, the backscattering intensity was analyzed owing to the non-transparency of the casting solutions. As delineated in Figure 9A-F1, the slowly increased backscattering data of all the heights as a function of time signifies the overall coalescence of the TiO_2_ nanoparticles throughout the casting solution sample for membrane F1. This result was consistent with the fact that the TiO_2_ nanoparticles were supposed to agglomerate along with the dissolution of the PVDF polymer in the organic solvent at 80 °C (indicated in Figure 9B-F1) [34]. The dynamic agglomeration of the TiO_2_ nanoparticles was ascribed to the fact that the high surface energy of the nanoparticles and the less viscous casting solution during the preparation process aggravated the instability of the casting solution, which agreed with the high *TSI* value (see Figure 10). Unlike the linetype of the casting solution sample for membrane F1, the scans of the casting solution sample for membrane F2 appeared as slightly “packed”, forming thick solid lines (see Figure 9A-F2). It can be confirmed that the presence of a certain quantity of homogeneous polymeric casting solution could enhance the stability of the final nano-composited casting solution during the preparation process. The overlapping scans for sample F3, indicating superior stability, might be attributed to the preferable pre-dispersion of TiO_2_ nanoparticles in the presence of small organic polymers, i.e., PEG. As shown in Figure 9B-F3, the hydrophilic interaction between the hydroxyl on the PEG and the TiO_2_ nanoparticles might have formed flower-like micelles; consequently, the steric hindrance effect derived from the PEG chains of flowerlike micelles might have benefited the stable dispersion of the TiO_2_ nanoparticles in the suspending organic solutions (in the pre-dispersing step) as well as the viscous final casting solutions [20]. The lowest *TSI* value (<1.0 for membrane F3) through the whole measuring period (shown in Figure 10) further validated this assumption. Based on our previous study, the gradually enhanced comprehensive performances, including hydrophilicity and antifouling behaviors, among others, might have benefited from the ascending stability of the casting solutions of membranes F1–F3 [32].

## 4. Conclusions

In this study, the influence of the dispersion status of TiO_2_ nanoparticles during the preparation process of a composite casting solution on the morphologies, properties and antifouling performance of PVDF/TiO_2_ composite membranes was systematically investigated. PEG was employed as a pre-dispersing agent in partial solvent aimed at pre-dispersing the TiO_2_ nanoparticles and, also in addition, as a pore-forming agent during the subsequent phase inversion process. The pre-dispersion of the TiO_2_ nanoparticles facilitated the formation of uniform-surface pores and the development of a finger-like structure on the sub-layers. The porosity and water permeability were improved differently. The hydrophilicity was improved by 6.0% in terms of the contact angle value. The zeta potential and mechanical properties were also enhanced. The pre-dispersion of the TiO_2_ nanoparticles also contributed to the decrease in roughness on the membrane surfaces. The decreasing negative free energy of cohesion and adhesion and increasing interaction energy (up to 0.43 KT) between the membrane surfaces and foulants, along with the less declined flux, confirmed the promising modification of the antifouling performance of the PVDF/TiO_2_ composite membrane. The excellent stability of the TiO_2_ nanoparticles in the casting solution of membrane F3 after the pre-dispersing process under the steric hindrance interaction of PEG was inferred from the overlapping backscattering signal and relatively low *TSI* value (<1.0), which might have given rise to the excellent comprehensive performance of the PVDF/TiO_2_ composite membrane.

## Figures and Tables

**Table 1:** Porosity, water permeability, contact angle and zeta potential of PVDF/TiO_2_ composite membranes F1–F3.

Membrane No.	Porosity (%)	Water Permeability (L/(m^2^·h·kPa)	Contact Angle (°)	Zeta Potential (mV)
F1	35.27 ± 3.71	62.91 ± 0.77	88.07 ± 0.97	−25.22 ± 1.83
F2	41.21 ± 4.67	65.74 ± 1.77	87.22 ± 0.23	−26.66 ± 0.36
F3	37.01 ± 4.67	64.96 ± 0.27	82.81 ± 0.51	−27.18 ± 0.33

**Table 2:** Mechanical properties of PVDF/TiO_2_ composite membranes F1–F3.

Membrane No.	Tensile Strength (MPa)	Elongation at Break (%)
F1	32.06 ± 0.68	17.65 ± 0.57
F2	33.00 ± 1.05	11.37 ± 0.35
F3	35.06 ± 1.74	18.45 ± 0.58

**Table 3:** Roughness of PVDF/TiO_2_ composite membranes F1–F3.

Membrane No.	*R*q (nm)	*R*a (nm)	*R*max (nm)
F1	25.97 ± 1.31	20.10 ± 1.18	295.50 ± 55.86
F2	22.65 ± 1.34	17.80 ± 0.99	246.00 ± 11.31
F3	26.30 ± 2.69	20.30 ± 1.13	245.00 ± 48.08

**Table 4:** Properties of PVDF/TiO_2_ composite membranes F1–F3 and BSA (*n* = 3).

BSA	Concentration (g/L)	pH	Size (nm)	
	1.0	7.0	322.9 ± 4.4	
Membrane/BSA	Zeta Potential (mV)	Contact Angle (°)		
		Water	Formamide	Diiodomethane
F1	−25.2 ± 1.8	88.1 ± 1.0	55.8 ± 1.1	47.2 ± 0.2
F2	−26.7 ± 0.4	87.2 ± 0.2	55.5 ± 0.1	48.0 ± 0.1
F3	−27.2 ± 0.3	82.8 ± 0.5	47.2 ± 0.7	42.2 ± 0.2
BSA	−10.3 ± 0.3	66.1 ± 2.4	52.7 ± 1.8	48.4 ± 2.0

**Table 5:** Surface tension parameters and surface free energy of PVDF/TiO_2_ composite membranes at the separation distance of *h*_0_ (0.157 ± 0.009 nm) (*n* = 3).

**Surface Tension Parameters for Each Membrane and BSA (mJ/m^2^)**						
Membrane NO.	*γ* ^LW^	*γ* ^+^	*γ* ^−^	*γ* ^AB^		*γ* ^TOT^
F1	35.82 ± 0.10	1.11 ± 0.11	0.75 ± 0.15	0.91 ± 0.12		36.73 ± 0.20
F2	35.37 ± 0.03	1.16 ± 0.03	0.98 ± 0.08	1.07 ± 0.03		36.44 ± 0.03
F3	38.50 ± 0.10	1.78 ± 0.09	1.09 ± 0.05	1.39 ± 0.06		39.89 ± 0.16
BSA	35.14 ± 1.13	0.28 ± 0.14	17.01 ± 1.87	2.14 ± 0.61		37.28 ± 0.79
	**The Free Energy of Cohesion of Membrane (mJ/m^2^)**			**The Free Energy of Adhesion of Membranes (mJ/m^2^)**		
Membrane NO.	Δ*G*_121_^LW^	Δ_121_*G*^AB^	Δ*G*_121_^SWS^	Δ*G*_123_^LW^	Δ*G*_123_^AB^	Δ*G*_123_^SWS^
F1	−3.47 ± 0.04	−66.97 ± 1.93	−70.43 ± 1.90	−3.31 ± 0.02	−45.43 ± 0.84	−48.75 ± 0.83
F2	−3.27 ± 0.01	−64.53 ± 0.48	−67.80 ± 0.49	−3.22 ± 0.01	−44.21 ± 0.34	−47.43 ± 0.34
F3	−4.72 ± 0.05	−59.57 ± 0.82	−64.28 ± 0.77	−3.87 ± 0.02	−43.25 ± 0.26	−47.12 ± 0.24

**Table 6:** Free energy of cohesion of PVDF/TiO_2_ composite membranes F1–F3 (*n* = 3) and free energy of adhesion between membrane surfaces and foulants (*n* = 3).

Membrane No.	Free Energy of Cohesion (mJ/m^2^)	Free Energy of Adhesion (mJ/m^2^)
F1	−70.43 ± 1.90	−48.75 ± 0.83
F2	−67.80 ± 0.49	−47.43 ± 0.34
F3	−64.28 ± 0.77	−47.12 ± 0.24

## Data Availability

The data presented in this study are available on request from the corresponding author.
